# Artificial selection on brain size leads to matching changes in overall number of neurons

**DOI:** 10.1111/evo.13805

**Published:** 2019-08-01

**Authors:** Lucie Marhounová, Alexander Kotrschal, Kristina Kverková, Niclas Kolm, Pavel Němec

**Affiliations:** ^1^ Department of Zoology, Faculty of Science Charles University 12844 Prague Czech Republic; ^2^ Behavioural Ecology Group Department of Animal Sciences 6708wd Wageningen Netherlands; ^3^ Department of Zoology/Ethology Stockholm University 10691 Stockholm Sweden

**Keywords:** Artificial selection, brain size, cognition, isotropic fractionator, number of neurons

## Abstract

Neurons are the basic computational units of the brain, but brain size is the predominant surrogate measure of brain functional capacity in comparative and cognitive neuroscience. This approach is based on the assumption that larger brains harbor higher numbers of neurons and their connections, and therefore have a higher information‐processing capacity. However, recent studies have shown that brain mass may be less strongly correlated with neuron counts than previously thought. Till now, no experimental test has been conducted to examine the relationship between evolutionary changes in brain size and the number of brain neurons. Here, we provide such a test by comparing neuron number in artificial selection lines of female guppies (*Poecilia reticulata*) with >15% difference in relative brain mass and numerous previously demonstrated cognitive differences. Using the isotropic fractionator, we demonstrate that large‐brained females have a higher overall number of neurons than small‐brained females, but similar neuronal densities. Importantly, this difference holds also for the telencephalon, a key region for cognition. Our study provides the first direct experimental evidence that selection for brain mass leads to matching changes in number of neurons and shows that brain size evolution is intimately linked to the evolution of neuron number and cognition.

The relationship between brain size and its functional capacity remains controversial. Several decades of comparative research on brain size variation in relation to body size have been based on the assumption that a larger brain also contains more neurons (Jerison 1973; Herculano‐Houzel 2017; Tsuboi et al. 2018) and it has been argued that it is the difference in neuron number that underlies the commonly found association between measures of brain size and cognitive abilities (McDaniel 2005; Kotrschal et al. 2013; MacLean et al. 2014; Benson‐Amram et al. 2016; Buechel et al. 2018; Horschler et al. 2019; Hwang et al. 2019).

The isotropic fractionator (Herculano‐Houzel and Lent 2005), a recent methodological breakthrough to quantify neuron numbers quickly and accurately (Bahney and von Bartheld 2014; Miller et al. 2014; Ngwenya et al. 2017), has now made it possible to test this assumption by quantifying how neuron numbers scale with brain mass both within and across species. This method involves mechanical dissociation of fixed brain tissue into a homogenous suspension of free cell nuclei, which are then counted and immunocytochemically identified to estimate the proportion of nonneuronal (glial and endothelial) cells and neurons (Herculano‐Houzel and Lent 2005). Data collected with this method have shown that similarly sized brains of vertebrates can differ in neuron number, neuronal densities, and allocation of neurons into different brain regions (Herculano‐Houzel et al. 2015a; Olkowicz et al. 2016). For instance, a primate brain accommodates many more neurons than a rodent brain of similar size (Herculano‐Houzel et al. 2007), and a parrot or songbird brain contains on average twice as many neurons as an equivalently sized primate brain (Olkowicz et al. 2016). These insights into differences in neuron numbers and densities offer a possible explanation as to why brain size sometimes does not predict cognitive ability, especially when comparison is made across distantly related species (Dicke and Roth 2016; Güntürkün and Bugnyar 2016). Moreover, neuronal density often shows a pattern of negative allometry with body and brain size across species (Herculano‐Houzel et al. 2015a; Olkowicz et al. 2016). Hence, small‐bodied species with smaller absolute brain size show higher neuronal densities than larger species with larger absolute brain size, although exceptions from this rule have been observed (Kverková et al. 2018). Whether this negative allometry pattern also exists within species or whether neuronal density is a species‐specific characteristic is currently unknown (but see Herculano‐Houzel et al. 2015b). When trying to understand the consequences of having a larger brain, it is therefore necessary to consider neuron number, and also ideally to test the effect of variation in brain size and neuron number on cognitive abilities.

The total number of neurons, elementary building blocks of the brain, is an important, though not the only determinant of brain information‐processing capacity. Other factors at play include the number of neuronal connections, neuron packing density, interneuronal distance, and axonal conduction velocity (Dicke and Roth 2016). Thus, besides the total number of neurons and their connections, diversification of neuronal types and their properties (Markram et al. 2015; Tasic et al. 2018; Tosches et al. 2018; Zeisel et al. 2018), and diversification of molecular machineries subserving neuronal signaling (Grant 2016; Zhu et al. 2018), all contribute to the broad behavioral repertoires seen in various vertebrates. Because quantitative mapping of cell type and synaptic density distributions across the brain is challenging and difficult to interpret, and because consequences of cellular and molecular diversification for cognitive processes remain poorly understood, here we focus on the number of neurons, which currently is the most feasible, easy‐to‐measure proxy for cognitive abilities. Areas controlling higher cognitive functions involve mainly telencephalic associative regions, which, in turn, rely on telencephalic sensorimotor and also cerebellar processing (Barton 2012). Indeed, a growing body of comparative evidence suggests that the absolute number of neurons in the telencephalon is a particularly good correlate of cognitive abilities (Dicke and Roth 2016; Olkowicz et al. 2016; Herculano‐Houzel 2017).

To investigate how evolutionary changes in brain size are related to changes in neuron number, we use artificial selection lines of guppies (*Poecilia reticulata*) that have been selected for relative brain size for five generations, resulting in >15 % differences in brain mass (Kotrschal et al. 2013). Importantly, several tests of various aspects of cognition in these selection lines have revealed substantial advantages of increased brain size in cognitive abilities, including numerical learning (Kotrschal et al. 2013), maze learning (Kotrschal et al. 2014), mate discrimination (Corral‐López et al. 2017a; Bloch et al. 2018), predator avoidance (Kotrschal et al. 2015; van der Bijl et al. 2015), and reversal learning (Buechel et al. 2018). At the same time, perception aspects, such as visual acuity (Corral‐López et al. 2017b), remained constant between the lines. Body size was not affected by artificial selection on relative brain size (Kotrschal et al. 2013, 2014). These selection lines thus offer the opportunity to test how evolutionary changes in brain size, demonstrably associated with changes in cognitive abilities, affect neuron number and neuron density independently of body size.

The vertebrate brain is divided into several regions with different functions and these regions can have strikingly different neuron numbers and densities (Herculano‐Houzel et al. 2015a; Olkowicz et al. 2016). It is therefore important to also examine differences in neuron numbers between key brain regions to get the complete picture. Importantly, the guppy lines used here and selected for large and small brains did not differ in relative volumes of 11 major brain regions (Kotrschal et al. 2017a). Here, we quantify numbers of neurons and nonneuronal (glial and endothelial) cells in the whole brain and the telencephalon of large‐ and small‐brained guppies using the isotropic fractionator. In a subset of individuals, we further quantify cell numbers in three other key brain regions. As these selection lines clearly differ in cognitive ability (see examples above), and we assume that more neurons provide higher computing power (Dicke and Roth 2016; Olkowicz et al. 2016; Herculano‐Houzel 2017), we expect that absolute (and relative) number of neurons in the brain and telencephalon will be higher in the large‐brained than in the small‐brained selection lines.

## Methods

### BRAIN SIZE SELECTION LINES

We quantified neuron number in 53 adult female guppies from the brain size selection lines (Kotrschal et al. 2013; Supporting Information Dataset S1). A total of 26 females originated from large‐brained selection lines, and 27 females from small‐brained selection lines. The selection regime consisted of three up‐selected and three down‐selected lines (see Kotrschal et al. 2013 for full description of the selection experiment). The individuals in this assay came from the fifth generation of selection and were adult virgin females. We focused on females in the study because most of the previous cognitive assays have been done on females.

### TISSUE PREPARATION

The fish were euthanized by an overdose of benzocaine and kept in 4% paraformaldehyde solution during transport from Stockholm University to Charles University in Prague, where body was weighted to the nearest 0.1 mg using a Kern ALJ (Kern & Sohn GmbH, Balingen‐Frommern, Germany) 120‐4 balance and standard body length (from the tip of the snout to the end of the caudal peduncle) was measured to the nearest 0.01 mm using an electronic digital calliper IP67. Immediately afterward, the brains were removed using an Olympus SZX (Olympus Corporation, Tokyo, Japan) 16 stereomicroscope and weighed to the nearest 0.001 mg using a Mettler Toledo (Mettler Toledo, Columbus, Ohio) MX5 microbalance. We divided the brains into two parts, the telencephalon and the “rest of the brain” comprising the diencephalon, tectum, cerebellum, and brainstem. To quantify neuron number and neuronal density in three additional brain regions, a subsample of 20 brains (11 and nine from the large‐ and small‐brained lines, respectively) were dissected into telencephalon, tectum (comprising the tectum opticum, torus semicircularis, and torus longitudinalis), cerebellum, and a division consisting of the brainstem and diencephalon. Cerebral hemispheres including the olfactory bulbs were detached from the rest of the brain by a transverse cut separating the telencephalon from the rostral pole of the tectum and diencephalon. The remaining brain was divided into left and right halves by a midsagittal cut. Subsequently, the cerebellum was excised from the surface of the brainstem together with the valvula cerebelli extending into the ventricle of the tectum. The tectum was then cut off from the remaining division, which consisted of the brainstem and the diencephalon. The latter more detailed dissections do not allow for statistical brain size selection line comparisons due to (1) small sample sizes and (2) potentially higher measurement errors as dissecting and homogenizing such small quantities is extremely challenging. Nevertheless, they provide an opportunity to coarsely characterize the guppy brain in numbers, at least for our study population. The final sample sizes for the different dissection protocols are given in Table 1. Immediately after dissection, all the brain divisions were weighed to the nearest 0.001 mg, and then kept in antifreeze solution (30% glycerol, 30% ethylene glycol, 40% phosphate buffer) at –25°C for later processing.

**Table 1:** Relative distribution of mass and cells in female guppy brain

Structure	Mass (mg)	Number of neurons	Neuronal density (N/mg)	Nonneuronal cells	Nonneuronal cells density (N/mg)	Glia/neurons ratio
Whole brain (*n* = 42)	4.8	4.3 × 10^6^	9.05 × 10^5^	2.2 × 10^6^	4.7 × 10^5^	0.52
	± 0.617	± 4.97 × 10^5^	± 8.9 × 10^4^	± 3.65 × 10^5^	± 7.6 × 10^4^	± 0.092
Telencephalon (*n* = 49)	0.83	6.33 × 10^5^	7.48 × 10^5^	3.93 × 10^5^	4.79 × 10^5^	0.65
	± 0.115	± 8.6 × 10^4^	± 8.3 × 10^4^	± 6.86 × 10^4^	± 7.1 × 10^4^	± 0.113
Tectum (*n* = 17)	1.36	1.1 × 10^6^	8.79 × 10^5^	6.31 × 10^5^	4.76 × 10^5^	0.55
	± 0.189	± 1.67 × 10^5^	± 1.4 × 10^5^	± 1.4 × 10^5^	± 1.08 × 10^5^	± 0.147
Cerebellum (*n* = 16)	0.48	1.7 × 10^6^	3.8 × 10^6^	4.3 × 10^6^	1 × 10^6^	0.27
	± 0.098	± 3.2 × 10^5^	± 6.89 × 10^5^	± 1.27 × 10^5^	± 3.799 × 10^5^	± 0.095
Diencephalon and brainstem	2.11	7.45 × 10^5^	3.56 × 10^5^	6.33 × 10^5^	2.9 × 10^5^	0.83
(*n* = 17)	± 0.291	± 1.25 × 10^5^	± 5.1 × 10^4^	± 1.54 × 10^5^	± 5.83 × 10^4^	± 0.224

### ISOTROPIC FRACTIONATOR METHODOLOGY

We estimated the total number of cells, neurons, and nonneuronal cells using the isotropic fractionator (Herculano‐Houzel and Lent 2005). Each dissected brain division was homogenized in 40 mM sodium citrate with 1% Triton X‐100 using Tenbroeck tissue grinders (0.5 mL, Ningbo Ja‐Hely Technology Co., Ltd., China). When turned into an isotropic suspension of isolated cell nuclei, the homogenate was transferred to an Eppendorf tube and the walls of the grinder were rinsed with dissociation solution to transfer all the cells to the tube. Then we measured the exact volume of the homogenate using an Eppendorf Xplorer (Eppendorf, Hamburg, Germany) 5–1000 µL electronic pipette and added a fluorescent DNA marker DAPI (4,6‐Diamidino‐2‐Phenylindole, Dihydrochloride) (5% of the total volume) to stain all nuclei. The total number of nuclei in suspension, and therefore the total number of cells in the original tissue, was estimated by determining the density of nuclei in small fractions drawn from the homogenate. At least six 10 µL aliquots were sampled and the number of cells was counted in a Neubauer‐improved counting chamber (BDH, Dagenham, Essex, UK) using an Olympus BX51 fluorescent microscope; additional aliquots (four to six) were counted when any coefficient of variation (CV) exceeded 0.05 (CV was always ≤ 0.10). After determining the total number of cells, the proportion of neurons was determined by immunocytochemical detection of neuronal nuclear marker NeuN (Mullen et al. 1992). We used rabbit polyclonal antibody anti‐NeuN (ABN78, dilution 1:800; Merck). The binding sites of the primary antibody were revealed by Alexa Fluor 594‐conjugated goat anti‐rabbit IgG (dilution 1:800; ThermoFisher Scientific). An electronic hematologic counter (Alchem Grupa, Torun, Poland) was used to count simultaneously DAPI‐labeled and NeuN‐immunopositive nuclei in the Neubauer chamber. A minimum of 500 nuclei were counted per sample to estimate percentage of double‐labeled neuronal nuclei. The number of nonneuronal cells was derived by subtracting the number of neurons from the total number of cells.

### DATA ANALYSIS

All statistical analyses were conducted in the R software environment version 3.5.1 (R Core Team 2018). As all dependent variables were normally distributed, we used linear mixed models (LMMs) implemented in the packages “lme4” (Bates et al. 2015) and “lmerTest” (Kuznetsova et al. 2017), with the trait of interest as the dependent variable, brain size selection treatment as a fixed effect (and, in case of relative measures, body/brain mass as a covariate), and replicate nested in brain size selection treatment as a random effect. We then selected the best model by stepwise backward elimination of nonsignificant effects, starting from the full model with an interaction between the covariate and selection treatment. In all cases, the interaction term was nonsignificant and removed from the model. Satterthwaite's approximation was used to estimate the effective degrees of freedom. Results are presented using the best‐fitting model parameters.

The Kendall's τ rank correlation coefficient was used to assess the association between brain tissue mass and the number of neurons and nonneuronal cells at the individual level using the base R package (R Core Team 2018).

### ETHICS

The experiment was performed in accordance with ethical applications approved by the Stockholm Ethical Board (Dnr: C50/12, N173/13, and 223/15).

## Results

### THE GUPPY BRAIN IN CELL NUMBERS

In this study, 53 females weighed between 247 and 580 mg, their brain mass ranged between 3.83 and 6.35 mg, and their brains contained between 3.35 and 5.73 million neurons (Table 1, Supporting Information Dataset S1). Whole brain neuron density ranged between 7.05 × 10^5^ and 1.76 × 10^6^ N/mg and was negatively associated with both body mass (LMM, *t*_1,40_ = 18.39, *R* ^2^ = 0.188, *P*  =  0.002) and brain mass (LMM, *t*_1,40_ = 14.97, *R* ^2^ = 0.302, *P* = <0.001). We found that neuron density varied greatly among the principal brain divisions examined. The highest average neuron density was detected in the cerebellum (∼3.82 × 10^6^ N/mg), and the lowest in the division comprising the diencephalon and brainstem (∼3.56 × 10^5^ N/mg). Consequently, different brain divisions harbored different amounts of neurons. The telencephalon constituted 17% of the brain mass and contained 15% of all brain neurons, the tectum constituted 28% of the brain mass and contained 26% of brain neurons, the brainstem and diencephalon together made up 44% of the brain mass but contained only 17% of brain neurons, and the densest brain region, the cerebellum, contained 40% of brain neurons despite representing only 10% of the total brain mass.

Nonneuronal (glial and endothelial) cells constituted a minor cellular fraction in all brain divisions (Table 1, Fig. S1A and S1B). Among individual females, the proportion of nonneuronal cells to neurons in the brain ranged between 27% and 44%. Hence, the maximum glia/neuron ratio (if all nonneuronal cells were glial cells) for the whole brain ranged between 0.36 and 0.77. Density of nonneuronal cells varied across brain regions, although to lesser extent than the density of neurons, and was loosely correlated with neuron density. We found the highest nonneuronal density in the cerebellum (∼1.03 × 10^6^ N/mg; Table 1), and the lowest in the division comprising the diencephalon and brainstem (∼2.91 × 10^5^ N/mg; Table 1).

### COMPARISON BETWEEN SELECTION LINES

**Figure 1:**
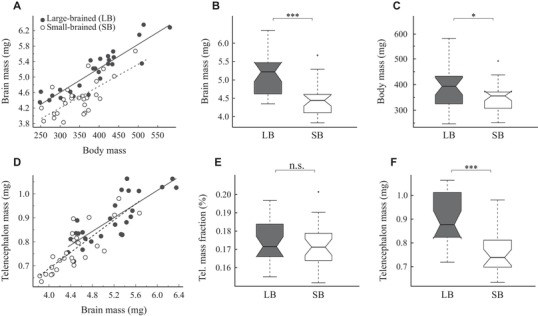
Brain and telencephalon size compared between small‐ and large‐brained selection lines. (A) Brain‐body scaling in female guppies. Note that allometric lines for small‐ and large‐brained guppies have significantly different intercepts, clearly indicating difference (grade shift) in relative brain mass (for statistics, see SI Results). Absolute brain mass (B) and body mass (C) compared between selection lines. (D) Telencephalon mass plotted as a function of brain mass. Note that relative mass of the telencephalon does not differ between the selection lines (for statistics, see Supporting Information Results). Telencephalon mass fraction (E) and absolute telencephalon mass (F) compared between selection lines. Each point in the scatterplots represents the values for one individual, the lines represent the ordinary least squares regressions for small‐brained (the dashed lines) and large‐brained (the solid lines) female guppies. Box plots denote median, 95% confidence intervals of median, first and third quartiles, total range, and outliers. The statistical significance level in box plots is indicated as follows: ^*^^*^^*^ *P* < 0.001; ^*^^*^ *P* < 0.01; ^*^ *P* < 0.05; n.s., nonsignificant). LB, large‐brained line; SB, small‐brained line.

We found that females from large‐ and small‐brained selection lines differed in both relative brain mass (LMM, *t*_3,50_ = 5.281, *P* < 10^−5^; Fig. 1A) and absolute brain mass (LMM, *t*_2,51_ = 4.804, *P* < 10^−4^; Fig. 1B), with females of large‐brained lines having approximately 15.4% larger brains compared to those of the small‐brained lines. Large‐brained females were also slightly heavier than small‐brained females (LMM, *t*_2,51_ = 2.09; *P* = 0.042; Fig. 1C).

**Figure 2:**
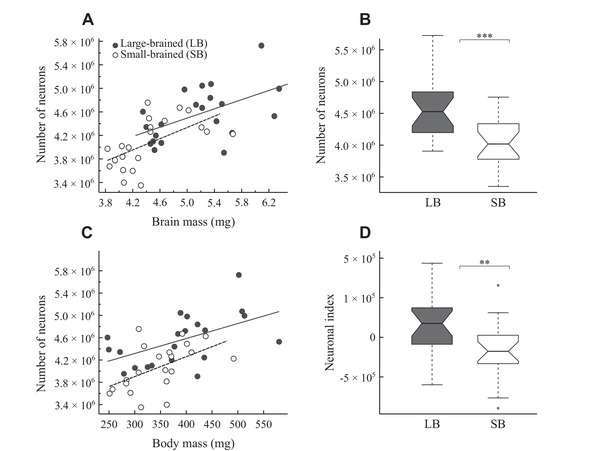
Neuronal scaling and neuron numbers compared between small‐ and large‐brained selection lines. (A) Number of neurons plotted as a function of brain mass. Note that the relationship between brain mass and the number of brain neurons does not differ between the selection lines (for statistics, see Supporting Information Results). (B) The total number of brain neurons compared between selection lines. (C) Number of neurons plotted as a function of body mass. Note that guppies of large‐brained line have significantly more neurons for a given body mass (for statistics, see Supporting Information Results). (D) Neuronal index (i.e., residuals from the neurons‐body regression line for all female guppies) compared between selection lines (see Figure 1 for explanation).

The relationship between brain mass and number of neurons could be described by similar linear functions in the large‐ and small‐brained selection lines (see Supporting Information Results and Table S1). Thus, the number of neurons relative to overall brain mass did not differ between the selection lines (LMM, *t*_3,39_ = 1.360, *P* = 0.182), meaning that large‐ and small‐brained lines showed similar neuronal densities (LMM, *P* = 0.292). However, due to their larger brains, the large‐brained lines had a higher total number of neurons than those of the small‐brained lines (LMM, *t*_2,40_ = 3.573, *P* < 0.001; Fig. 2B). This amounted to an 11.9% difference in neuron number between selection lines. To control for body size, we also examined residuals from a neuron number versus body mass regression to determine the “neuronal index” (Herculano‐Houzel 2007) and found a significantly higher neuronal index in large‐ compared to small‐brained individuals (LMM, *t*_2,40_ = 2.906, *P* < 0.006; Fig. 2D).

**Figure 3:**
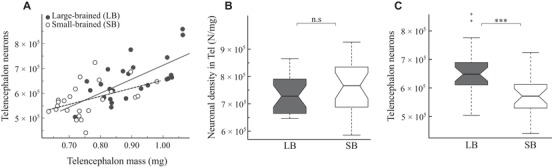
Neuronal scaling, densities, and numbers in the telencephalon. (A) Number of telencephalic neurons plotted as a function of telencephalon mass. Note that the relationship between telencephalon mass and number of telencephalic neurons does not differ between the selection lines (for statistics, see Supporting Information Results). (B and C) Neuronal densities in the telencephalon (B) and absolute number of telencephalic neurons (C) compared between selection lines (see Figure 1 for explanation).

Telencephalon mass correlated tightly with brain mass (LMM, *t*_2,51_ = 12.833, *P* < 10^−15^, *R* ^2^  = 0.76; Fig. 1D) but the telencephalon mass fraction did not differ between the lines (LMM, *P* = 0.521; Fig. 1E). Likewise, the number of telencephalic neurons correlated with telencephalon mass (LMM, *t*_2,47_ = 6.537, *P* < 10^−7^, *R* ^2^  = 0.47; Fig. 3A), but neuron density in the telencephalon did not differ between the lines (LMM, *P* = 0.203; Fig. 3B). In absolute terms, the telencephalon of the large‐brained lines was heavier (LMM, *t*_2,51_ = 4.756, *P* < 10^−4^; Fig. 1F) and harbored more neurons than the telencephalon of the small‐brained lines (LMM, *t*_2,47_ = 3.670, *P* < 0.001; Fig. 3B). Although the sample size for additional brain regions was too small to allow formal statistical comparisons of selection lines, we found qualitatively similar differences between the selection lines for all other brain regions, except the brain stem (Table S2).

The scaling of brain mass and number of nonneuronal cells did not differ between the selection lines (see Supporting Information Results, Fig. S1C). Likewise, no difference was observed in glia/neuron ratio (LMM, *P* ≥ 0.683 in all cases). But fish from the large‐brained lines tended to have higher absolute numbers of nonneuronal cells compared to fish from the small‐brained lines, although the difference was only significant for the telencephalon (LMM, telencephalon: *t*_2,47_ = 2.297, *P* = 0.026; whole brain: *t*_2,40_  = 1.773, *P* = 0.084; Fig. S1D).

### INDIVIDUAL DIFFERENCES IN CELLULAR DENSITIES

**Figure 4:**
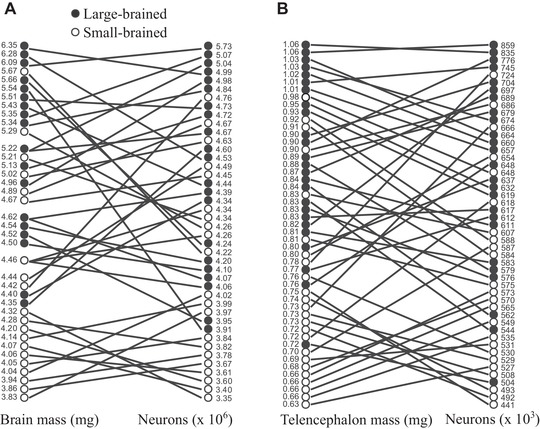
Individual differences in brain and telencephalon size, neuron numbers, and densities. Relationship between brain mass (A), telencephalon mass (B), and neuron counts. These variables are ranked in descending order from the largest to the smallest and individual values are given on the sides of the graphs. Solid lines connect values measured in the same individual. Crossed lines indicate individual differences in neuronal densities.

Apart from variation in brain mass, the mass of the examined brain regions and number of neurons and nonneuronal cells (see above), we also observed considerable individual differences in densities of neurons and nonneuronal cells (Fig. 4 and Fig. S2). Thus, although we detected clear differences between the brain size selection lines, individuals with the largest brains did not necessarily have the most neurons and/or nonneuronal cells. Nevertheless, the rank correlation between brain tissue mass and number of neurons was significant both in the whole brain (Kendall's τ = 0.46, *P* < 0.001; Fig. 4A) and in the telencephalon (Kendall's τ = 0.49, *P* < 0.001; Fig. 4B). The same pattern was observed for the association between brain tissue mass and number of nonneuronal cells (whole brain: Kendall's τ = 0.29, *P* = 0.007, Fig. S2A; the telencephalon: Kendall's τ = 0.39, *P* < 0.001, Fig. S2B). The observed individual differences in neuronal densities were much larger than expected measurement error (see Methods section), therefore it is unlikely that they represent mere technical artifacts.

## Discussion

Our results show that selection for larger and smaller brains also generates a matching increase in number of neurons. Moreover, the number of neurons increases linearly with increasing brain mass in the selection lines. The implications of this result are manifold. First, it suggests that brain mass can be an appropriate predictor of neuron number, at least at the within species level. It is important to acknowledge that the correlation between brain mass and neuron number was not very strong due to pronounced variation in neuronal densities. Yet, brain mass accounted for 47% of the observed variation in the number of neurons. This finding, however, should be generalized with caution as a much weaker correlation between brain mass and number of neurons has been observed in laboratory mice (Herculano‐Houzel et al. 2015b) and captive‐bred Madagascar ground geckos *Paroedura picta* (our own unpubl. data). This difference might be attributable to the relaxed selective pressure in captive‐bred populations. It is well known that animals bred in captivity often have smaller brains and behave differently than their wild counterparts (e.g., Price 1999; Kruska 2007; Guay and Iwaniuk 2008; Burns et al. 2009; LaDage et al. 2016; Jensen 2017). The mice and geckos were likely kept under low cognitive pressure, whereas guppies in this study were subjected to strong artificial selection on brain size. We hypothesize that the association between brain mass and neuron number might be stronger in wild populations. Natural selection in the wild acts on behavior, not on brain size, as the artificial selection performed here. Thus, individual variation in neuronal density in wild populations inhabiting the same selective environment might be decreased due to directional selection and therefore lower than that observed in captive populations. On the other hand, changes in the environment can trigger substantial changes in brain region size (for review, see Kotrschal et al. 2017b; Kotrschal et al. 2012; Gonda et al. 2013; Fong et al. 2019) and potentially also region‐specific changes in neuronal numbers and densities. Local control and variation in cell proliferation or survival may facilitate mosaic brain evolution in wild populations, when favored by selection (Montgomery et al. 2016). Assessment of neuronal density variation in natural populations is required to test these hypotheses.

Second, the remarkable finding that a 12% difference in neuron numbers arose within just five generations of artificial selection for brain size have important implications. It suggests that selection on individuals with more neurons or larger brains within a population can be an important microevolutionary mechanism underlying the evolution of brain size and information processing capacity, at least at the population and species level. This result also shows that such evolutionary changes can be very fast. It is notable in this context that two or three generations of guppies per year occur in the wild (Houde 1997; Magurran 2005).

Third, the evidence that the large‐brained guppies also have higher cognitive abilities (Kotrschal et al. 2013, 2014, 2015; van der Bijl et al. 2015; Corral‐López et al. 2017a; Bloch et al. 2018; Buechel et al. 2018) supports the idea that the number of neurons, either absolute or in relation to body size, is an important factor contributing to cognitive abilities. We propose that it is indeed the higher number of neurons in the larger brains in these selection lines that have yielded their cognitive advantages, especially because the differences in neuron number between large‐ and small‐brained females were consistent across whole brain and the telencephalon. It is worth noting that the large‐brained fish in this study were slightly larger than the small‐brained fish. This is the first time this has been encountered in more than 20 comparisons of body mass that have been done on subsamples of these selection lines, and the most likely explanation is therefore that we randomly picked differently sized individuals from the brain size selection lines. Importantly, the differences in neuron number between the large‐ and small‐brained lines were substantial and robust also when the analyses controlled for body mass.

Fourth, and finally, the observed neuronal scaling rules, that is, the relationship between brain mass or brain region mass and number of neurons (Herculano‐Houzel et al. 2006), were very similar in the large‐ and small‐brained lines. Despite the observed decreasing neuronal densities with increasing brain mass across all our animals and a slightly smaller body mass in the small‐brained lines, there were no significant differences in neuronal density between the brain size lines. Hence, it is not cellular composition but rather brain size that sets the large‐brained lines apart from the small‐brained lines. This supports what we have previously shown for brain region volumes (Kotrschal et al. 2017a), namely that the brains of the large‐brained lines are scaled‐up versions of the brains of the small‐brained lines. This is similar to what has been shown when comparing the large human brain to smaller nonhuman primate brains (Herculano‐Houzel 2009), and the large corvid brains to smaller non‐corvid songbird brains (Olkowicz et al. 2016). Hence, it seems that these mentioned differences in neuron numbers are generated mainly by the relative (and absolute) size of the brain.

Apart from the comparisons between the guppy brain size selection lines, we provide the first quantification of neuron number in the guppy brain. Although our quantification is done on selected laboratory populations, this makes it the second fish species with a known number of neurons and the first one with a known neuron count and known densities in specific brain regions. An extremely miniaturized cyprinid fish *Danionella translucida* from Myanmar has the smallest known adult vertebrate brain possessing only 650 thousand neurons (Schulze et al. 2018), which is almost eight times less than reported here for the guppy. By contrast, the zebrafish *Danio rerio* seems to have a higher number of brain neurons, because its brain contains on average 36% more cells (Hinsch and Zupanc 2007). All these small fish have tiny brains and extremely high neuronal densities. For instance, whole brain neuron densities reported here for the guppy are approximately twofold and approximately 4.4‐fold higher than the highest whole brain neuronal densities reported in birds and mammals, respectively (Sarko et al. 2009; Olkowicz et al. 2016). These observations confirm a trend found in other taxa (Herculano‐Houzel et al. 2015a; Olkowicz et al. 2016), namely that smaller bodied species with correspondingly smaller brains have higher neuron density than larger species with larger brains. We suggest that this is one mechanism to compensate for the small absolute brain size in small‐bodied vertebrates that have to solve relatively complex ecological and social problems in their natural environments. As already mentioned, neuron numbers in most brain regions examined matched the relative size of the region with the exception of the cerebellum, which showed an almost fourfold higher neuron number than expected for its size. This is a similar pattern as found in birds and mammals (Herculano‐Houzel 2010; Herculano‐Houzel et al. 2015a; Olkowicz et al. 2016). Just like in other vertebrates (for review, see Barton 2012; Baumann et al. 2014; Sokolov et al. 2017), the cerebellum in teleost fishes is important for many functions such as motor coordination and movement but also cognitive processes (Kotrschal et al. 1998; Butler and Hodos 2005; Rodríguez et al. 2005; Braithwaite 2006; Kolm et al. 2009; Gómez et al. 2010; Warren and Sawtell 2016). The high absolute numbers of cerebellar neurons thus indicate that this region is important in computationally demanding tasks also in the guppy. The neuron richness of the cerebellum highlights the need to estimate neuron numbers and not just region size to fully appreciate brain region functional capacity (for further discussion, see Herculano‐Houzel 2010; Barton 2012).

The density of nonneuronal cells was also high in the population of guppies studied here. Depending on the brain region and taxon, they are two to four times higher than the highest nonneuronal cell densities reported in birds and mammals (Sarko et al. 2009; Herculano‐Houzel et al. 2015a; Olkowicz et al. 2016; Dos Santos et al. 2017; Kocourek et al. unpubl. data). It remains unclear whether the high nonneuronal cell densities in the guppy represent the corollary of miniaturization or a feature that is shared by all teleost fishes. Interestingly, the degree of variation in nonneuronal cell density across different brain regions in our fish is comparable to that of birds and mammals but generally much less pronounced than variation in densities of neurons (Herculano‐Houzel et al. 2015a; Olkowicz et al. 2016). Although these findings indicate that nonneuronal scaling rules are much more conserved than neuronal scaling rules, they weaken the notion that nonneuronal densities are largely independent of brain size, brain region, and taxon investigated (Herculano‐Houzel et al. 2014; Olkowicz et al. 2016).

To conclude, we demonstrate that selection for brain size in the guppy has generated matching changes in the number of neurons, and these differences are similar across the whole brain and the telencephalon, a key region for cognition. We also show that neuronal density scales negatively with brain size at the intraspecific level, replicating previous findings across species in other taxa. Importantly, together with earlier studies assessing behavior in large‐ and small‐brained guppies (see above), this study provides the first direct demonstration of a close association between brain size, neuron numbers, and cognitive abilities at the intraspecific level. Thus, our findings provide experimental support for the idea that neuron numbers adequately predict cognitive abilities.

Associate Editor: A. M. Rice

Handling Editor: Mohamed A. F. Noor

## Supporting information


**Table S1**. Scaling rules for female guppy brains from brain size selection lines. Power laws were calculated from the individual values listed in dataset S1.
**Table S2**. Relative distribution of mass and cells in female guppy brains from brain size selection lines. G/N ratio, glia to neuron ratio.
**Figure S1**. Glia/neuron ratios, nonneuronal cell scaling, and numbers compared between small‐ and large‐brained selection lines.
**Figure S2**. Individual differences in brain and telencephalon size, nonneuronal cell numbers, and densities.
Guppies dataset.

## References

[evo13805-bib-0001] Bahney, J. , and C. S. von Bartheld . 2014 Validation of the isotropic fractionator: Comparison with unbiased stereology and DNA extraction for quantification of glial cells. J. Neurosci. Methods 222:165–174. 24239779 10.1016/j.jneumeth.2013.11.002 PMC3904371

[evo13805-bib-0002] Barton, R. A. 2012 Embodied cognitive evolution and the cerebellum. Philos. Trans. R. Soc. B 367:2097–2107. 10.1098/rstb.2012.0112 PMC3385677 22734053

[evo13805-bib-0003] Bates, D. , M. Maechler , B. Bolker , S. Walker , R. H. B. Christensen , H. Singmann , B. Dai , F. Scheipl , G. Grothendieck , P. Green , et al. 2015 Package ‘lme4’. Convergence 12:1.

[evo13805-bib-0004] Baumann, O. , R. J. Borra , J. M. Bower , K. E. Cullen , C. Habas , R. B. Ivry , M. Leggio , J. B. Mattingley , M. Molinari , E. A. Moulton , et al . 2014 Consensus paper: The role of the cerebellum in perceptual processes. Cerebellum 14:197–220. 10.1007/s12311-014-0627-7 PMC4346664 25479821

[evo13805-bib-0005] Benson‐Amram, S. , B. Dantzer , G. Stricker , E. M. Swanson , and K. E. Holekamp . 2016 Brain size predicts problem‐solving ability in mammalian carnivores. Proc. Natl. Acad. Sci. USA 113:2532–2537. 26811470 10.1073/pnas.1505913113 PMC4780594

[evo13805-bib-0006] Bloch, N. I. , A. Corral‐López , S. D. Buechel , A. Kotrschal , N. Kolm , and J. E. Mank . 2018 Early neurogenomic response associated with variation in guppy female mate preference. Nat. Ecol. Evol. 2:1772–1781. 30297748 10.1038/s41559-018-0682-4 PMC6349141

[evo13805-bib-0007] Braithwaite, V. A. 2006 Cognitive ability in fish Pp. 1–37 *in* Sloman K., Balshine S., and Wilson R., eds. Behaviour and physiology of fish, vol. 24 Associated Press, San Diego, CA.

[evo13805-bib-0008] Buechel, S. D. , A. Boussard , A. Kotrschal , W. van der Bijl , and N. Kolm . 2018 Brain size affects performance in a reversal‐learning test. Proc. R. Soc. B 285:20172031. 10.1098/rspb.2017.2031 PMC5805926 29367391

[evo13805-bib-0009] Burns, J. G. , A. Saravanan , F. Helen Rodd . 2009 Rearing environment affects the brain size of guppies: Lab‐reared guppies have smaller brains than wild‐caught guppies. Ethology 115:122–133.

[evo13805-bib-0010] Butler, A.B. , and W. Hodos . 2005 Comparative vertebrate neuroanatomy: Evolution and adaptation. John Wiley & Sons, Hoboken, NJ.

[evo13805-bib-0011] Corral‐López, A. , N. I. Bloch , A. Kotrschal , W. van der Bijl , S. D. Buechel , J. E. Mank , and N. Kolm . 2017a Female brain size affects the assessment of male attractiveness during mate choice. Sci. Adv. 3:e1601990. 28345039 10.1126/sciadv.1601990 PMC5362185

[evo13805-bib-0012] Corral‐López, A. , M. Garate‐Olaizola , S. D. Buechel , N. Kolm , and A. Kotrschal . 2017b On the role of body size, brain size, and eye size in visual acuity. Behav. Ecol. Sociobiol. 71:179. 10.1007/s00265-017-2408-z PMC5705735 29213179

[evo13805-bib-0013] Dicke, U. , and G. Roth . 2016 Neuronal factors determining high intelligence. Philos. Trans. R. Soc. B 371:20150180. 10.1098/rstb.2015.0180 PMC4685590 26598734

[evo13805-bib-0014] Dos Santos, S. E. , J. Porfirio , F. B. da Cunha , P. R. Manger , W. Tavares , L. Pessoa , M. A. Raghanti , C. C. Sherwood , and S. Herculano‐Houzel . 2017 Cellular scaling rules for the brains of marsupials: Not as ‘primitive’ as expected. Brain Behav. Evol. 89:48–63. 28125804 10.1159/000452856

[evo13805-bib-0016] Fong, S. , Buechel, S. D. , Boussard, A. , Kotrschal, A. , & Kolm, N. 2019 Plastic changes in brain morphology in relation to learning and environmental enrichment in the guppy (*Poecilia reticulata*). J. Exp. Biol. 222 10.1242/jeb.200402 31053644

[evo13805-bib-0017] Gonda, A. , G. Herczeg , and J. Merilä . 2013 Evolutionary ecology of intraspecific brain size variation: A review. Ecol. Evol. 3:2751–2764. 24567837 10.1002/ece3.627 PMC3930043

[evo13805-bib-0018] Gómez, A. , E. Durán , C. Salas , and F. Rodríguez . 2010 Cerebellum lesion impairs eyeblink‐like classical conditioning in goldfish. Neuroscience 166:49–60. 20006973 10.1016/j.neuroscience.2009.12.018

[evo13805-bib-0019] Grant, S. G. 2016 The molecular evolution of the vertebrate behavioural repertoire. Philos. Trans. R Soc. Lond. B Biol. Sci. 371:20150051. 26598730 10.1098/rstb.2015.0051 PMC4685586

[evo13805-bib-0020] Guay, P. J. , and A. N. Iwaniuk . 2008 Captive breeding reduces brain volume in waterfowl (Anseriformes). Condor 110:276–284.

[evo13805-bib-0021] Güntürkün, O. , and T. Bugnyar . 2016 Cognition without cortex. Trends Cogn. Sci. 20:291–303. 26944218 10.1016/j.tics.2016.02.001

[evo13805-bib-0022] Herculano‐Houzel, S. 2007 Encephalization, neuronal excess, and neuronal index in rodents. Anat. Rec. 290:1280–1287. 10.1002/ar.20598 17847061

[evo13805-bib-0023] Herculano‐Houzel, S. 2009 The human brain in numbers: a linearly scaled‐up primate brain. Front. Hum. Neurosci. 3:31. 19915731 10.3389/neuro.09.031.2009 PMC2776484

[evo13805-bib-0024] Herculano‐Houzel, S. 2010 Coordinated scaling of cortical and cerebellar numbers of neurons. Front. Neuroanat. 4:12. 20300467 10.3389/fnana.2010.00012 PMC2839851

[evo13805-bib-0025] Herculano‐Houzel, S. 2017 Numbers of neurons as biological correlates of cognitive capability. Curr. Opin. Behav. Sci. 16:1–7.

[evo13805-bib-0026] Herculano‐Houzel, S. , and R. Lent . 2005 Isotropic fractionator: A simple, rapid method for the quantification of total cell and neuron numbers in the brain. J. Neurosci. 25:2518–2521. 15758160 10.1523/JNEUROSCI.4526-04.2005 PMC6725175

[evo13805-bib-0027] Herculano‐Houzel, S. , B. Mota , and R. Lent . 2006 Cellular scaling rules for rodent brains. Proc. Natl. Acad. Sci. USA 103:12138–12143. 16880386 10.1073/pnas.0604911103 PMC1567708

[evo13805-bib-0028] Herculano‐Houzel, S. , C. E. Collins , P. Wong , and J. H. Kaas . 2007 Cellular scaling rules for primate brains. Proc. Natl. Acad. Sci. USA 104:3562–3567. 17360682 10.1073/pnas.0611396104 PMC1805542

[evo13805-bib-0029] Herculano‐Houzel, S. , P. R. Manger , and J. H. Kaas . 2014 Brain scaling in mammalian evolution as a consequence of concerted and mosaic changes in numbers of neurons and average neuronal cell size. Front. Neuroanat. 8:77. 25157220 10.3389/fnana.2014.00077 PMC4127475

[evo13805-bib-0030] Herculano‐Houzel, S. , K. Catania , P. R. Manger , and J. H. Kaas . 2015a Mammalian brains are made of these: A dataset of the numbers and densities of neuronal and nonneuronal cells in the brain of Glires, Primates, Scandentia, Eulipotyphlans, Afrotherians and Artiodactyls, and their relationship with body mass. Brain Behav. Evol. 86:145–163. 26418466 10.1159/000437413

[evo13805-bib-0031] Herculano‐Houzel, S. , D. J. Messeder , K. Fonseca‐Azevedo , and N. A. Pantoja . 2015b When larger brains do not have more neurons: Increased numbers of cells are compensated by decreased average cell size across mouse individuals. Front. Neuroanat. 9:64. 26082686 10.3389/fnana.2015.00064 PMC4450177

[evo13805-bib-0032] Hinsch, K. , and G. K. H. Zupanc . 2007 Generation and long‐term persistence of new neurons in the adult zebrafish brain: A quantitative analysis. Neuroscience 146:679–696. 17395385 10.1016/j.neuroscience.2007.01.071

[evo13805-bib-0033] Horschler, D. J. , B. Hare , J. Call , J. Kaminski , Á. Miklósi , and E. L. MacLean . 2019 Absolute brain size predicts dog breed differences in executive function. Anim. Cogn. 22:187–198. 30607673 10.1007/s10071-018-01234-1

[evo13805-bib-0034] Houde, A. 1997 Sex, color, and mate choice in guppies. Princeton Univ. Press, Princeton, NJ.

[evo13805-bib-0035] Hwang, L. D. , L. T. Strike , B. Couvy‐Duchesne , G. I. de Zubicaray , K. McMahon , P. A. S. Breslin , D. R. Reed , N. G. Martin , and M. J. Wright . 2019 Associations between brain structure and perceived intensity of sweet and bitter tastes. Behav. Brain Res. 363:103–108. 30703394 10.1016/j.bbr.2019.01.046 PMC6470356

[evo13805-bib-0036] Jensen, P. 2017 The ethology of domestic animals: An introductory text. Cabi, Boston, MA.

[evo13805-bib-0037] Jerison, H. J. 1973 Evolution of the brain and intelligence. Academic Press, New York, NY.

[evo13805-bib-0039] Kolm, N. , A. Gonzalez‐Voyer , D. Brelin , and S. Winberg . 2009 Evidence for small scale variation in the vertebrate brain: Mating strategy and sex affect brain size and structure in wild brown trout (*Salmo trutta*). J. Evol. Biol. 22:2524–2531. 19878498 10.1111/j.1420-9101.2009.01875.x

[evo13805-bib-0040] Kotrschal, A. , Sundström, L.F. , Brelin, D. , Devlin, R.H. , Kolm, N. 2012 Inside the heads of David and Goliath: Environmental effects on brain morphology among wild and growth‐enhanced coho salmon, *Oncorhynchus kisutch* . J. Fish Biol. 81:987–1002. 22880732 10.1111/j.1095-8649.2012.03348.x

[evo13805-bib-0041] Kotrschal, K. , M. J. Van Staaden , and R. Huber . 1998 Fish brains: Evolution and environmental relationships. Rev. Fish Biol. Fisher. 8:373–408.

[evo13805-bib-0042] Kotrschal, A. , B. Rogell , A. Bundsen , B. Svensson , S. Zajitschek , I. Brännström , S. Immler , A. A. Maklakov , and N. Kolm . 2013 Artificial selection on relative brain size in the guppy reveals costs and benefits of evolving a larger brain. Curr. Biol. 23:168–171. 23290552 10.1016/j.cub.2012.11.058 PMC3566478

[evo13805-bib-0043] Kotrschal, A. , A. Corral‐Lopez , M. Amcoff , and N. Kolm . 2014 A larger brain confers a benefit in a spatial mate search learning task in male guppies. Behav. Ecol. 26:527–532. 25825587 10.1093/beheco/aru227 PMC4374130

[evo13805-bib-0044] Kotrschal, A. , S. D. Buechel , S. M. Zala , A. Corral‐Lopez , D. J. Penn , and N. Kolm . 2015 Brain size affects female but not male survival under predation threat. Ecol. Lett. 18:646–652. 25960088 10.1111/ele.12441 PMC4676298

[evo13805-bib-0045] Kotrschal, A. , H.‐L. Zeng , W. van der Bijl , C. Öhman‐Mägi , K. Kotrschal , K. Pelckmans , and N. Kolm . 2017a Evolution of brain region volumes during artificial selection for relative brain size. Evolution 71:2942–2951. 28986929 10.1111/evo.13373

[evo13805-bib-0046] Kotrschal, A. , A. E. Deacon , A. E. Magurran , and N. Kolm . 2017b Predation pressure shapes brain anatomy in the wild. Evol. Ecol. 31:619–633. 10.1007/s10682-017-9901-8 PMC6961500 32009719

[evo13805-bib-0047] Kruska, D. 2007 The effect of domestication on brain size Pp. 143–153 *in* Kaas J. H., ed. Evolution of nervous system. A comprehensive reference. Academic Press, New York, NY.

[evo13805-bib-0048] Kuznetsova, A. , P. B. Brockhoff , R. H. B. Christensen . 2017 lmerTest package: Tests in linear mixed effects models. J. Stat. Softw. 82 10.18637/jss.v082.i13

[evo13805-bib-0049] Kverková, K. , T. Bělíková , S. Olkowicz , Z. Pavelková , M. J. O'Riain , R. Šumbera , H. Burda , N. C. Bennett , and P. Němec . 2018 Sociality does not drive the evolution of large brains in eusocial African mole‐rats. Sci. Rep. 8:9203. 29907782 10.1038/s41598-018-26062-8 PMC6003933

[evo13805-bib-0050] LaDage, L. D. , T. C. Roth II, B. Sinervo , and V. V. Pravosudov . 2016 Environmental experiences influence cortical volume in territorial and nonterritorial side‐blotched lizards, Uta stansburiana. Anim. Behav. 115:11–18.

[evo13805-bib-0051] MacLean, E. L. , B. Hare , C. L. Nunn , E. Addessi , F. Amici , R. C. Anderson , F. Aureli , J. M. Baker , A. E. Bania , A. M. Barnard , et al. 2014 The evolution of self‐control. Proc. Natl. Acad. Sci. USA 111:E2140–E2148. 24753565 10.1073/pnas.1323533111 PMC4034204

[evo13805-bib-0052] Magurran, A. E. 2005 Evolutionary ecology: The Trinidadian guppy. Oxford Univ. Press, Oxford, U.K.

[evo13805-bib-0053] Markram, H. , E. Muller , S. Ramaswamy , M. W. Reimann , M. Abdellah , C. A. Sanchez , A. Ailamaki , L. Alonso‐Nanclares , N. Antille , S. Arsever , et al. 2015 Reconstruction and simulation of neocortical microcircuitry. Cell 163: 456–492. 26451489 10.1016/j.cell.2015.09.029

[evo13805-bib-0054] McDaniel, M. 2005 Big‐brained people are smarter: A meta‐analysis of the relationship between in vivo brain volume and intelligence. Intelligence 33:337–346.

[evo13805-bib-0055] Miller, D. J. , P. Balaram , N. A. Young , and J. H. Kaas . 2014 Three counting methods agree on cell and neuron number in chimpanzee primary visual cortex. Front. Neuroanat. 8:36. 24904305 10.3389/fnana.2014.00036 PMC4032965

[evo13805-bib-0056] Montgomery, S. H. , N. I. Mundy , and R. A. Barton . 2016 Brain evolution and development: Adaptation, allometry and constraint. Proc. R. Soc. B 283:20160433. 10.1098/rspb.2016.0433 PMC5031648 27629025

[evo13805-bib-0057] Mullen, R. J. , C. R. Buck , and A. M. Smith . 1992 NeuN, a neuronal specific nuclear protein in vertebrates. Development 116(1):201–211. 1483388 10.1242/dev.116.1.201

[evo13805-bib-0058] Ngwenya, A. , J. Nahirney , B. Brinkman , L. Williams , and A. N. Iwaniuk . 2017 Comparison of estimates of neuronal number obtained using the isotropic fractionator method and unbiased stereology in day old chicks (*Gallus domesticus*). J. Neurosci. Methods 287:39–46. 28587893 10.1016/j.jneumeth.2017.05.025

[evo13805-bib-0059] Olkowicz, S. , M. Kocourek , R. K. Lučan , M. Porteš , W. T. Fitch , S. Herculano‐Houzel , and P. Němec . 2016 Birds have primate‐like numbers of neurons in the forebrain. Proc. Natl. Acad. Sci. USA 113:7255–7260. 27298365 10.1073/pnas.1517131113 PMC4932926

[evo13805-bib-0060] Price, E. O. 1999 Behavioral development in animals undergoing domestication. Appl. Anim. Behav. Sci. 65:245–271.

[evo13805-bib-0061] Rodríguez, F. , E. Durán , A. Gómez , F. M. Ocaña , E. Álvarez , F. Jiménez‐Moya , C. Broglio , and C. Salas . 2005 Cognitive and emotional functions of the teleost fish cerebellum. Brain Res. Bull. 66:365–370. 16144616 10.1016/j.brainresbull.2004.11.026

[evo13805-bib-0062] Sarko, D. K. , K. C. Catania , D. B. Leitch , J. H. Kaas , and S. Herculano‐Houzel . 2009 Cellular scaling rules of insectivore brains. Front. Neuroanat. 3:8. 19636383 10.3389/neuro.05.008.2009 PMC2713736

[evo13805-bib-0063] Sokolov, A. A. , R. C. Miall , and R. B. Ivry . 2017 The cerebellum: Adaptive prediction for movement and cognition. Trends Cogn. Sci. 21:313–332. 28385461 10.1016/j.tics.2017.02.005 PMC5477675

[evo13805-bib-0064] R Core Team. 2018 R: A language and environment for statistical computing. R Foundation for Statistical Computing, Vienna.

[evo13805-bib-0065] Schulze, L. , J. Henninger , M. Kadobianskyi , T. Chaigne , A. I. Faustino , N. Hakiy , S. Albadri , M. Schuelke , L. Maler , F. Del Bene , et al . 2018 Transparent *Danionella translucida* as a genetically tractable vertebrate brain model. Nat. Methods 15:977–983. 30323353 10.1038/s41592-018-0144-6

[evo13805-bib-0066] Tasic, B. , Z. Yao , L. T. Graybuck , K. A. Smith , T. N. Nguyen , D. Bertagnolli , J. Goldy , E. Garren , M. N. Economo , S. Viswanathan , et al. 2018 Shared and distinct transcriptomic cell types across neocortical areas. Nature 563(7729):72. 30382198 10.1038/s41586-018-0654-5 PMC6456269

[evo13805-bib-0067] Tosches, M. A. , T. M. Yamawaki , R. K. Naumann , A. A. Jacobi , G. Tushev , and G. Laurent . 2018 Evolution of pallium, hippocampus, and cortical cell types revealed by single‐cell transcriptomics in reptiles. Science 360:881–888. 29724907 10.1126/science.aar4237

[evo13805-bib-0068] Tsuboi, M. , W. van der Bijl , B. T. Kopperud , J. Erritzøe , K. L. Voje , A. Kotrschal , K. E. Yopak , S. P. Collin , A. N. Iwaniuk , and N. Kolm . 2018 Breakdown of brain–body allometry and the encephalization of birds and mammals. Nat. Ecol. Evol. 2:1492–1500. 30104752 10.1038/s41559-018-0632-1

[evo13805-bib-0069] van der Bijl, W. , M. Thyselius , A. Kotrschal , and N. Kolm . 2015 Brain size affects the behavioural response to predators in female guppies (Poecilia reticulata). Proc. Biol. Sci. B 282:20151132. 10.1098/rspb.2015.1132 PMC4528528 26203003

[evo13805-bib-0070] Warren, R. , and N. B. Sawtell . 2016 A comparative approach to cerebellar function: Insights from electrosensory systems. Curr. Opin. Neurobiol. 41:31–37. 27504860 10.1016/j.conb.2016.07.012 PMC5123925

[evo13805-bib-0071] Zeisel, A. , H. Hochgerner , P. Lönnerberg , A. Johnsson , F. Memic , J. Van der Zwan , M. Häring , E. Braun , L. E. Borm , G. La Manno , et al. 2018 Molecular architecture of the mouse nervous system. Cell 174:999–1014. 30096314 10.1016/j.cell.2018.06.021 PMC6086934

[evo13805-bib-0072] Zhu, F. , M. Cizeron , Z. Qiu , R. Benavides‐Piccione , M. V. Kopanitsa , N. G. Skene , B. Koniaris , J. DeFelipe , E. Fransén , N. H. Komiyama , et al. 2018 Architecture of the mouse brain synaptome. Neuron. 99:781–799. 30078578 10.1016/j.neuron.2018.07.007 PMC6117470

