# Supplementary material for: Artificial selection on brain size leads to matching changes in overall number of neurons
Source: Evolution. 2019 Aug 1;73(9):2003–12. doi: 10.1111/evo.13805 (PMC6772110; doi:10.1111/evo.13805)
Supplement: Supplementary file 1 — Table S1. Scaling rules for female guppy brains from brain size selection lines. Power laws were calculated from the individual values listed in dataset S1. Table S2. Relative distribution of mass and cells in female guppy brains from brain size selection lines. G/N ratio, glia to neuron ratio. Figure S1. Glia/neuron ratios, nonneuronal cell scaling, and numbers compared between small‐ and large‐brained selection lines. Figure S2. Individual differences in brain and telencephalon size, nonneuronal cell numbers, and densities. Guppies dataset. [file EVO-73-2003-s001.zip › evo13805-sup-0003-SuppMat.docx]

**Supporting material**

**Results**

**Comparison of mass and cellular scaling rules between the small-brained and the large-brained selection lines**

Regression lines for the small-brained and the large-brained selection lines are compared using generalized linear mixed models. The results of the analysis are summarized below for selected, important comparisons and listed in order, in which they appear in the figures.

**ad figure 1.** (a) Allometric lines for the small-brained and the large-brained guppies have the same slopes (LMM, F_3,50_ = 0.214, p = 0.646) but significantly different intercepts (F_3,50_ = 27.886, p < 10^-5^), indicating that females of large-brained line have significantly larger brains for a given body mass.

(d) Allometric lines for the small-brained and the large-brained guppies do not differ from each other (slopes: F_3,50_ = 0.688, p = 0.411; intercepts: F_3,50_ = 1.399, p = 0.243), indicating that relative mass of the telencephalon does not differ between the selection lines.

**ad figure 2.** (a) Allometric lines for the small-brained and the large-brained guppies do not differ from each other (slopes: F_3,39_ = 0.172, p = 0.681; intercepts: F_3,39_ = 1.180, p = 0.182), indicating that the relationship between brain mass and number of brain neurons does not differ between the selection lines.

(c) Allometric lines for the small-brained and the large-brained guppies do not differ in slopes (LMM, F_3,39_ = 0.277, p = 0.602) but they do significantly differ in intercepts (F_3,39_ = 8.875, p = 0.005), indicating that guppies of large-brained line have significantly more neurons for a given body mass.

**ad figure 3.** (a) Allometric lines for the small-brained and the large-brained guppies do not differ from each other (slopes: F_3,46_ = 1.550, p = 0.220; intercepts: F_3,46_ = 0.604, p = 0.441), indicating that the relationship between telencephalon mass and number of telencephalic neurons does not differ between the selection lines.

**ad figure S1.** (c) Allometric lines for the small-brained and the large-brained guppies do not differ from each other (slopes: F_3,39_ = 1.219, p = 0.276; intercepts: F_3,39_ = 0.141, p = 0.709), indicating that the relationship between brain mass and number of nonneuronal cells does not differ between the selection lines.

**Supplementary figures**

**Figure S1. Glia/neuron ratios, nonneuronal cell scaling and numbers** **compared between small- and large-brained selection lines.** (a, b) Proportion of nonneuronal cells (left axis) and the glia/neuron ratio (right axis) plotted against brain mass. (a) The overall glia/neuron ratio in the brain. (b) Glia/neuron ratio in the telencephalon and the rest of brain. (c) Number of nonneuronal cells plotted as a function of brain mass. Note that the relationship between brain mass and number of nonneuronal cells does not differ between the selection lines (for statistics, see SI Results). (d) Total number of nonneuronal cells in the brain compared between selection lines. Each point in the scatterplots represents the values for one individual. The lines in (c) represent the ordinary least squares regressions for small-brained (the dashed line) and large-brained (the solid line) female guppies. Box plots denote median, 95% confidence intervals of median, first and third quartiles, and total range. LB, large-brained line; n.s., non-significant; SB, small-brained line.

**Figure S2. Individual differences in brain and telencephalon size, nonneuronal cell numbers and densities.** (a, b) Relationship between brain mass (a), telencephalon mass (b) and nonneuronal cell counts. These variables are ranked in descending order from the largest to the smallest and individual values are given on the sides of the graphs. Solid lines connect values measured in the same individual. Crossed lines indicate individual differences in nonneuronal cell densities.

**Supplementary tables**

**Table S1**. Scaling rules for female guppy brains from brain size selection lines. Power laws were calculated from the individual values listed in dataset S1. BR, brain; BO, body; M, mass (in milligrams); N, number of neurons; O, number of other (nonneuronal) cells; r2, coefficient of determination calculated from the reduced major axis regression; TEL, telencephalon.

| Line | Dependent variable | Independent variable | Linear function | R^2^ | P value |
| --- | --- | --- | --- | --- | --- |
| Small-brained | M_BR_ | M_BO_ | M_BR_ = 0.00603 × M_BO_ + 2.378 | 0.78 | < 0.001 |
|  | N_BR_ | M_BR_ | N_BR_ = 500201 × M_BR_ + 1912509 | 0.43 | < 0.001 |
|  | N_BR_ | M_BO_ | N_BR_ = 2958 × M_BO_ + 3043396 | 0.42 | < 0.001 |
|  | M_TEL_ | M_BR_ | M_TEL_ = 0.16237 × M_BR_ + 0.04946 | 0.76 | <0.001 |
|  | N_TEL_ | M_TEL_ | N_TEL_ = 499905 × M_TEL_ + 201184 | 0.47 | <0.001 |
|  | O_BR_ | M_BR_ | O_BR_ = 230017 × M_BR_ + 1135145 | 0.15 | 0.005 |
|  | O_TEL_ | M_TEL_ | O_TEL_ = 326859 × M_TEL_ + 125664 | 0.31 | <0.001 |
| Large-brained | M_BR_ | M_BO_ | M_BR_ = 0.00603 × M_BO_ + 2.813 | 0.78 | < 0.001 |
|  | N_BR_ | M_BR_ | N_BR_ = 500201 × M_BR_ + 1912509 | 0.43 | < 0.001 |
|  | N_BR_ | M_BO_ | N_BR_ = 2958 × M_BO_ + 3405395 | 0.42 | < 0.001 |
|  | M_TEL_ | M_BR_ | M_TEL_ = 0.16237 × M_BR_ + 0.04946 | 0.76 | <0.001 |
|  | N_TEL_ | M_TEL_ | N_TEL_ = 499905 × M_TEL_ + 201184 | 0.47 | <0.001 |
|  | O_BR_ | M_BR_ | O_BR_ = 230017 × M_BR_ + 1135145 | 0.15 | 0.005 |
|  | O_TEL_ | M_TEL_ | O_TEL_ = 326859 × M_TEL_ + 125664 | 0.31 | <0.001 |

**Table S2.** Relative distribution of mass and cells in female guppy brains from brain size selection lines. G/N ratio, glia to neuron ratio.

| Line | Structure | Mass (mg) | Number of neurons | Neuronal  density (N/mg) | Nonneuronal cells | Nonneuronal cells density | G/N ratio |
| --- | --- | --- | --- | --- | --- | --- | --- |
| Large-brained | **Whole brain (n = 26**) | 5.15  ± 0.58 | 4.55 ×10^6^  ± 4.5 × 10^5^ | 8.91 × 10^5^  ± 9.1 × 10^4^ | 2.3 × 10^6^  ± 3.8 × 10^5^ | 4.5 × 10^5^  ± 8.3 × 10^4^ | 0.519  ± 0.1 |
|  | **Telencephalon (n = 25)** | 0.89  ± 0.1 | 6.55 × 10^5^  ± 8.4 × 10^4^ | 7.32 × 10^5^  ± 7.1 × 10^4^ | 4.17 × 10^5^  ± 6.1 × 10^4^ | 4.54 × 10^5^  ± 5.0 × 10^4^ | 0.64  ± 0.1 |
|  | **Optic tectum (n = 9)** | 1.46  ± 0.15 | 1.23 × 10^6^  ± 1.8 × 10^5^ | 8.65 × 10^5^  ± 1.76 × 10^5^ | 6.11 × 10^5^  ± 1.47 × 10^5^ | 4.19 × 10^5^  ± 7.47 × 10^4^ | 0.51  ± 0.15 |
|  | **Cerebellum**  **(n = 9)** | 0.50  ± 0.11 | 1.8 × 10^6^  ± 3.6 × 10^5^ | 3.85 × 10^6^  ± 6.27 × 10^5^ | 5.0 × 10^5^  ± 1.4 × 10^5^ | 1.09 × 10^6^  ± 4.89 × 10^5^ | 0.28  ± 0.11 |
|  | **Diencephalon and brainstem (n = 11)** | 2.25  ± 0.22 | 7.47 × 10^5^  ± 9.5 × 10^4^ | 3.33 × 10^5^  ± 4.5 × 10^4^ | 6.43 × 10^5^  ± 1.77 × 10^5^ | 2.89 × 10^5^  ± 7.31 × 10^4^ | 0.88  ± 0.27 |
| Small-brained | **Whole brain (n = 27)** | 4.46  ± 0.45 | 4.07 × 10^6^  ± 4.17 × 10^5^ | 9.2 × 10^5^  ± 8.6 × 10^4^ | 2.14 × 10^6^  ± 3.3 × 10^5^ | 4.84 × 10^5^  ± 6.79 × 10^4^ | 0.527  ± 0.07 |
|  | **Telencephalon (n = 24)** | 0.77  ± 0.09 | 5.74 × 10^5^  ± 6.74 × 10^4^ | 7.6 × 10^5^  ± 9.3 × 10^4^ | 3.74 × 10^5^  ± 6.97 × 10^4^ | 4.96 × 10^5^  ± 7.8 × 10^4^ | 0.657  ± 0.13 |
|  | **Optic tectum (n = 8)** | 1.24  ± 0.15 | 1.08 × 10^5^  ± 1.08 × 10^5^ | 8.94 × 10^5^  ± 9.6 × 10^4^ | 6.51 × 10^5^  ± 1.39 × 10^5^ | 5.39 × 10^5^  ± 1.08 × 10^5^ | 0.61  ± 0.13 |
|  | **Cerebellum**  **(n = 7)** | 0.45  ± 0.07 | 1.62 × 10^6^  ± 2.31 × 10^5^ | 3.79 × 10^6^  ± 8.1 × 10^5^ | 4.14 × 10^5^  ± 8.7 × 10^4^ | 9.49 × 10^5^  ± 1.64 × 10^5^ | 0.26  ± 0.08 |
|  | **Diencephalon and brainstem (n = 6)** | 1.94  ± 0.29 | 7.6 × 10^5^  ± 1.78 × 10^5^ | 3.96 × 10^5^  ± 3.5 × 10^4^ | 5.57 × 10^5^  ± 9.8 × 10^4^ | 5.6 × 10^5^  ± 9.8 × 10^4^ | 0.74  ± 0.08 |
