# Supplementary figures and images for: Artificial selection on brain size leads to matching changes in overall number of neurons
Source: Evolution. 2019 Aug 1;73(9):2003–12. doi: 10.1111/evo.13805 (PMC6772110; doi:10.1111/evo.13805)

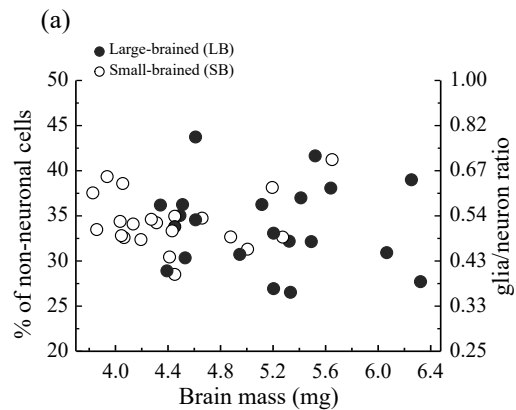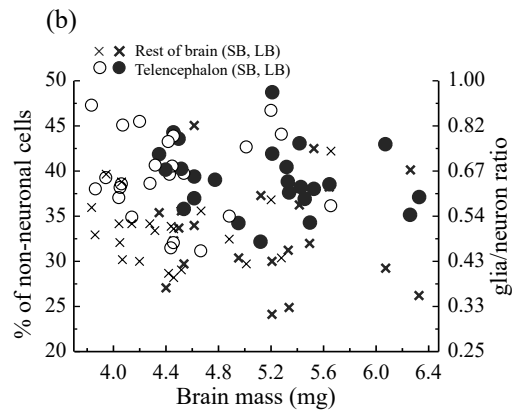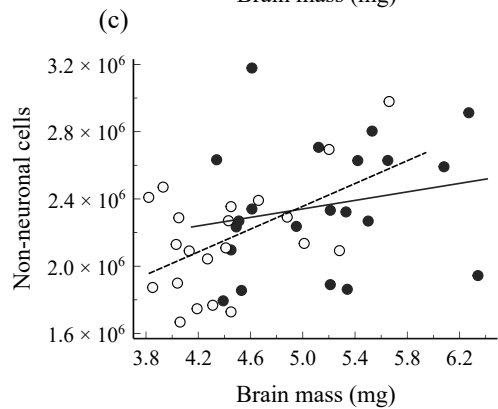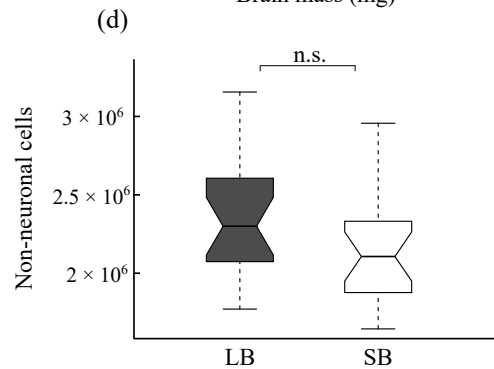

Supplement: Supplementary file 1 — Table S1. Scaling rules for female guppy brains from brain size selection lines. Power laws were calculated from the individual values listed in dataset S1. Table S2. Relative distribution of mass and cells in female guppy brains from brain size selection lines. G/N ratio, glia to neuron ratio. Figure S1. Glia/neuron ratios, nonneuronal cell scaling, and numbers compared between small‐ and large‐brained selection lines. Figure S2. Individual differences in brain and telencephalon size, nonneuronal cell numbers, and densities. Guppies dataset. [file EVO-73-2003-s001.zip › evo13805-sup-0001-FigureS1.pdf.pdf]

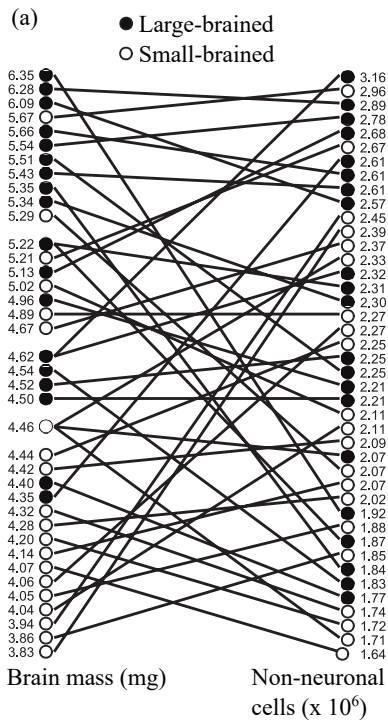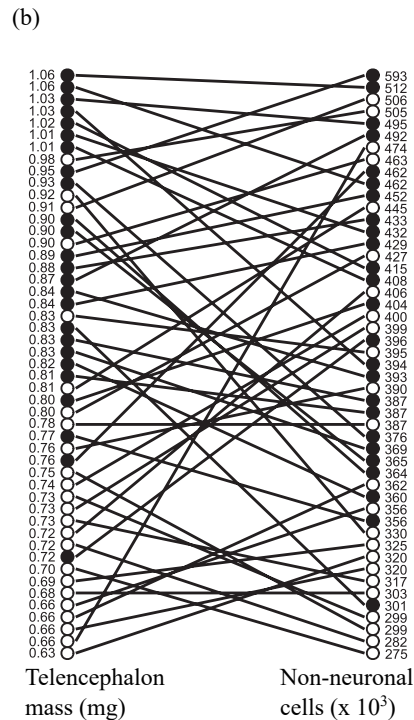

Supplement: Supplementary file 1 — Table S1. Scaling rules for female guppy brains from brain size selection lines. Power laws were calculated from the individual values listed in dataset S1. Table S2. Relative distribution of mass and cells in female guppy brains from brain size selection lines. G/N ratio, glia to neuron ratio. Figure S1. Glia/neuron ratios, nonneuronal cell scaling, and numbers compared between small‐ and large‐brained selection lines. Figure S2. Individual differences in brain and telencephalon size, nonneuronal cell numbers, and densities. Guppies dataset. [file EVO-73-2003-s001.zip › evo13805-sup-0002-FigureS2.pdf.pdf]
